# Address at the Opening of the Session for 1854–55 in the Medical Department of the University of Louisville

**Published:** 1854-12

**Authors:** Austin Flint

**Affiliations:** Professor of the Theory and Practice of Medicine in the University of Louisville


### MEDICINE AND SURGERY.

DECEMBER, 1854. NEW SERIES. VOL. II.—No. 12.

### ART. I.—ADDRESS AT THE OPENING OF THE SESSION FOR 1854–55 IN THE MEDICAL DEPARTMENT OF THE UNIVERSITY OF LOUISVILLE.

BY AUSTIN FLINT, M. D., PROEESSOR OF THE THEORY AND PRACTICE OF MEDICINE IN THE UNIVERSITY OF LOUISVILLE.

OBEDIENCE to a time-honored custom requires that the beginning of each successive term of instruction in our medical colleges shall be signalized by a public address, and the discharge of this duty on the occasion of the opening of the session for 1854–5 in this institution has been entrusted to me.

These introductory discourses have been so long repeated, and so often given to the medical public by means of the press, that it is at length usually felt to be a matter of some difficulty to find topics which have not been already abundantly discussed. In the present instance circumstances have relieved me of an embarrassment on this score, which, otherwise, I should assuredly have experienced. It is known to many of those whom I address, that at the close of the course of lectures in this college for 1853–4, I took my departure for Europe, remaining abroad until shortly before the commencement of the preliminary course just finished. The greater part of the period of my absence was spent in Paris. After leaving Paris I found time only to visit the capitols of England, Scotland and Ireland, making a brief stay in each of these cities.

It will probably be expected that the topics of my remarks on this occasion will have reference to medical matters that interested me during my late sojourn on the other side of the Atlantic. It would be, of course, impossible within the limits of an introductory discourse to undertake any thing like a comprehensive account of my observations and experiences, or to engage in any elaborate discussions. I propose, simply, in a desultory way, to recal some of the facts which arrested my attention, and the impressions which I received, indulging, in connection therewith, necessarily in terms very brief, a few reflections suggested by a comparative view of American medicine.

I shall confine myself to medical matters in Paris, in part because my personal observations there were more extended, but also because, so far as relates to the interests of medical science and education, the provisions in France may be assumed to be unquestionably superior to those in Great Britain. To assume this, however, is by no means to disparage the zeal and success with which medicine is cultivated and taught in London, Edinburg, and Dublin. Moreover, to extend my remarks to the latter places would require more time than I could venture to occupy, on an occasion like the present.

Aside from special objects which may constitute the chief inducement to visit foreign countries, the medical traveller, first of all, is desirous to see distinguished men with whose scientific labors he has been long familiar. And it is curious, in many instances, to compare the notions which had been previously formed of their appearance and manners with the reality. The ideal portraits of individuals, which the mind receives through the media of books, often contrasts singularly with representations as they appear to the eye. Moreover, contrary to the laws of optics, when great men are viewed in imagination, their magnitude is apt to be inversely in proportion to the shortness of focal distance. To endeavor to measure the real dimensions of magnates in our profession by inspection, may thus afford encouragement to the modest observer, and enhance his self-reliance. Herein consists one source of profit in extending to other lands the circle of our acquaintance. Another advantage also, may be derived, in addition to the gratification of a natural curiosity. After having seen, and still more, after personal interviews with those who hold conspicuous positions, one feels better qualified to place a correct estimate on the degree of consideration and confidence to which their opinions, and the results of their labors are entitled.

The visitor at Paris, whose professional life extends backward a quarter of a century, finds still living and working in the hospitals, the academies and the school of medicine, not a few of those who were eminent at the time when his retrospections commence. At the single hospital of *La Charité*, during the past summer, might be seen each morning, between the hours of 7 o'clock and 10, VELPEAU, the veteran surgeon of world wide fame; RAYER, author of an imperishable work on diseases of the skin, published in 1826; CRUVEILHIER, whose classical treatises, with splendid illustrations, on anatomy, healthy and morbid, albeit their age, are still standard authorities; PIORRY, whose labors in behalf of physical diagnosis followed closely on the discovery of auscultation by LAENNEC; BOUILLAUD, whose valuable contributions to our knowledge of diseases of the heart were anterior to those of HOPE; ANDRAL, whose publications on clinical medicine date as far back as the days of BROUSSAISM, and BRIQUET whose recent work on Quinia is to be added to other publications which have long been before the profession. Here is a corps of distinguished representatives of "Old Physic," with whose names those of us to whom, in this country, such an appellation is sometimes applied, were familiar in the days of our medical pupilage; and there they are not only maintaining their preëminence, but holding their ground as active working-men in the field of medical science.

This illustrates a fact which cannot fail to impress an American observer in Europe, viz: that age, wealth, honors, and the highest official position are considered in no degree inconsistent with continued application to scientific pursuits. Ease and retirement, the *ignis fatuus* of an *otium cum dignitate* do not entice from the paths of industry. To die in harness appears to be coveted in preference to a living death of inaction and imbecility. So died ROUX, a few days before my arrival at Paris, in the language of an eulogist, "pen in hand," leaving unfinished, "the scientific monument destined to crown his long and laborious career," a work entitled "forty years of Surgery."

I was struck, both in France and Great Britain, with the contrast presented to my eyes between the actual and apparent ages of most of the distinguished members of our profession whom I had the good fortune to see. Old men exhibited few of the evidences of declining years, and men of middle age retained the aspect of youth. Of the medical staff at *la Charité*, for example, VELPEAU, CRUVEILHIER and ANDRAL have reached an advanced period of life, yet their movements scarcely evince an approach of decrepitude, and in the discharge of their duties they do not show diminished quicness or vigor of intellect. When in London, I witnessed a surgical operation at St. Bartholomew's Hospital by LAWRENCE, performed with as much grace and dexterity, apparently, as if he were only in the meridian of life. Men who have long been distinguished, like RAYER, CAZENAVE, JOBERT, in Paris; CHRISTISON in Edinburg; STOKES in Dublin, seem to be in the prime of manhood; and others whom I could mention, the date of whose career of distinction is later, but still far from recent, we should class, from their appearance, among the youthful members of the profession. A signal instance of the latter occurs to me in the person of Dr. WALSHE, of London.

As regards the comparative duration of life, in Europe and America, of members of the medical profession, I am not prepared to form an opinion. Statistical dates adequate to decide this point, so far as I know, are wanting. But that the external manifestations of accumulated years, as a general remark, are less conspicuous than with us, and the body, if not the powers of the mind, maintained in a better state of preservation in spite of the influences of declining age, I think cannot be doubted. The causes of this difference are probably to be found in the custom, in this country, to engage earlier in active professional duties, to incur sooner domestic responsibilities, and in a variety of ways to guard with less care against circumstances involving wear and tear of the physical and mental constitution.

For the last twenty-five years, or more, Paris has been generally considered to possess greater facilities for pursuits connected with medical science, than any other city in the world. Students and medical men from widely separated quarters of the globe, have been attracted thither in greater numbers than at any other point.

Of late there has been some diversion in favor of other places, such as Berlin, Vienna, Giessen and Dublin; but the supremacy of Paris is still maintained. Fashion and the power of example have something to do, doubtless, in determining this influx from different countries. Aside from these influences, the attractions have been derived from the reputation of the Parisian teachers, the great resources for practical instruction, the wise policy with which the French government has instituted provisions for medical education and the promotion of medical science, and the liberality with which all the advantages of the metropolis are thrown open to persons of every nation, creed and complexion.

Instruction in every department of knowledge is provided at the University of France without expense to the recipient. The twenty-six professors constituting the faculty of medicine in Paris (as well as the professors in all the other departments,) receive stated salaries, and are in no measure dependent for their emolument on the number of their auditors. Moreover, until the establishment of the present Empire, every medical office, from the hospital *interne*, upward, was filled by means of a system of competition called the *concours*—a system designed to obviate favoritism, and secure success to the most meritoreous aspirants. The *concours* consists in a public trial as respects talent, scientific attainment, and skill in composition and speaking, made before a judicial board composed of members from the corps of professors. A candidate adjudged by the board to have given evidence of the greatest degree of fitness for the office competed for, receives the appointment. This, in the abstract, appears to be perfectly fair, and truly democratic. It is, however, a matter of notoriety that appointments have been obtained by extrinsic influences brought to bear on the judicial board; and it is well understood that certain persons, whatever amount of evidence they might give of fitness, must expect to fail from having rendered themselves obnoxious to a majority of the *faculty*. It has been sometimes proposed to transplant to this country the system of *concours* in the appointment of medical teachers; but we have already, virtually, such a system, more effective and satisfactory in its operation, than would be a closer imitation of the method pursued in France. As medical institutions are constituted in this country, any one whose attainments and character afford reasonable ground for the belief that he will prove a successful teacher is almost sure to have an opportunity to demonstrate what he can do in that capacity; and in proportion as he is found to possess the elements of success, will he be called to occupy positions of honor and responsibility, whenever vacancies occur. With some exceptional instances, the competition for places in American medical schools is as fair as that existing in France. The very best security for this is the fact that the character and success of our medical schools depend mainly on their obtaining the best teachers that they can command. Moreover, the fact that the continued prosperity of institutions in this country depends chiefly on the efforts of their faculties, in connection with the fact that the emolument of the professors is determined solely by the amount of this prosperity, furnishes incentives to exertion, and a safeguard against relaxation of zeal and energy, which would not be expected to be operative to the same extent were the permanency of incumbents, and the compensation altogether independent, as in France, of the medical classes.

The system of *concours* has recently been abolished in its application to the appointment of professors, the present Emperor having appropriated the power of filling vacancies as they occur. It still remains in force, however, with respect to all other medical offices.

It is not to be inferred from the fact of instruction in France being gratuitous, that a medical degree is obtained without expense. The medical student, during his four years of pupilage, is obliged to register his name every three months at the *bureau* of the medical faculty. For each of these *inscriptions*, as they are called, sixteen in number, a fee is required. He is also obliged to pay a fee for each of the five examinations to which he is subjected at different periods of his pupilage; for the examination and printing of his thesis, for the study of practical anatomy, and for his diploma, the aggregate amounting to about $250, a sum somewhat greater than is demanded for two courses of instruction at the most expensive of American colleges. I am led to make this statement because it appears to be a current belief that a medical education in Paris costs nothing, and the gratuitous instruction in France is sometimes cited as a precedent for the action of certain schools in this country, which have adopted a policy dictated by the supposition that their interests will be promoted by offering to students the temptation of low fees.

The medical student in France is under no obligation to attend the lectures given by the faculty. No evidence of his having listened to any of the professors is required for graduation. The inscriptions and satisfactory examinations, together with certain preliminary qualifications, among which are included a degree of batchelor of letters, or, in lieu thereof, a satisfactory examination in Latin and Greek, are alone necessary. This being the case, the several courses provided by the University are numerously attended, or otherwise, in proportion to the popularity of the teacher, and they are neglected entirely by the student if he thinks he can derive greater advantage from extra official instruction, and is willing to pay the small fees generally required for the latter.

The number of persons present at the different lectures given in succession at the school of medicine, varies greatly. Some of the professors draw a full house, others moderately, and in some instances the number of hearers is quite insignificant. I was present at a lecture by ADELON, on legal, or forensic medicine, when there were not more than a dozen in attendance. The lecture was entirely written, and read in a low monotonous tone of voice, the lecturer, much of the time, sitting with one elbow resting on the table, the hand supporting his head. Extra-official, or private lectures are also, of course, patronized in proportion to the reputation of the teacher, the importance of the subject, and the facilities for instruction at his command. The smallest audience which I saw assembled, was at a clinical lecture by M. TESSIER, a homœopothist, who has succeeded, thus far, since his profession of the creed of Hahnemann, in holding a situation as one of the attending physicians of the hospital Beaujon. On that occasion there were three medical students present! This may be regarded as a fair commentary on the estimation in which homœopathy is held at the present moment in Paris.

The lectures by the medical faculty of Paris are all given at the school of medicine, in the same lecture room. For the anatomical and chemical, as well as all the other courses, no other place is provided. The lectures which succeed each other follow with scarcely any intermission, and, hence, owing to the change of auditory, the beginning and end of each lecture are not a little disturbed by the ingress and egress of a greater or less number of persons. In comfort and elegance their common lecture-room will not compare with the average of those in American colleges. The walls are dingy with smoke and dirt; the benches are rough and without backs. In the latter particular the French have exhibited their usual tact, the construction of the seats being well calculated to obviate the loss of consciousness, if not to induce attention on the part of those in attendance. In all the hospital amphitheatres, and private lecture-rooms, plain, backless, and not over neat benches only are provided, a fact which may be commended to the consideration of those who sometimes express dissatisfaction with *our* comparatively luxurious accommodations, being solicitous, it is presumed, for still greater facilities for somnolency!

With very few exceptions, the professors are seated during the delivery of their lectures. This accords with the style of lecturing which is almost uniformly purely didactic. Contrary to what might be expected from the ardent temperament of the French people, and the amount of gesticulation usually exhibited by their public speakers, the scientific lectures are delivered in a quiet, colloquial manner, without any effort at rhetorical or oratorical display. The lecturer generally engages at once in his subject; very rarely indulges in any digressions, or episodes; seldom takes pains to relieve the attention of his hearers by an amusing anecdote, and at the expiration of his hour abruptly closes. None of the lecturers that I heard were deficient in ease and fluency, but they rarely exhibited animation or enthusiasm in their manner of delivery. The clinical lectures by TROUSSEAU, at the Hôtel Dieu, were more marked by the latter traits than those of any other teacher to whom I listened. The lectures by the distinguished physiologist BERNARD, which were beautifully lucid, and intensely interesting, might be said to be singularly modest as respects the traits just named, in view of the fact that they were in a great measure devoted to an exposition of the results of his own experimental observations, results which have contributed, within the few past years, to change materially the aspect of physiological science.

As regards the mutual relations of professor and pupil, I saw nothing to provoke criticism. The demeanor of the classes in the lecture-room, and at the hospitals, was considerate and respectful. No exceptions to this rule of conduct came under my notice. On the other hand, the bearing of the teachers was uniformly courteous, and free alike from a degree of reserve amounting to coldness, and an affectation of dignity repelling approach.

A concourse of medical students in Paris presents a motley collection. A variety of countries are represented, England, America, Spain, Portugal, Turkey, Sweden, the German States, and even the African race is not without its sable delegates. The diversity of costume is not less than of physiognomy. In external indications of intellect I do not hesitate to say that a class of American students would in no respects suffer by comparison. And as regards habits of application, from all that I could learn, the result of such a comparison would be decidedly in favor of the latter. The French medical students, as I was credibly informed, are not, in general, distinguished for devotion to pursuits pertaining to medical science, save in so far as is requisite to fit themselves to pass the prescribed examinations. I make this statement on the authority of one of the most eminent of the *agrégés*, or assistant professors of the University, and it accords with what I learned from several sources. The intervals between lectures, by a large proportion, are habitually spent at *cafès*, and the various places of amusement with which the French metropolis abounds. The private courses of lectures and demonstrations, constituting the most valuable portion of the educational advantages which Paris offers, are in a great measure sustained by the foreigners resorting to Paris for professional improvement. I trust it may be long ere a comparison is even admissible between the majority of medical students in Paris and America, in refinement and moral habits. In this point of view the former have a notoriety even in the public opinion of Paris, which is very far from enviable.

The preëminent position which Paris has so long held as a field for instruction and research in medical science, is generally attributed, in a great measure, to the extent of its clinical resources and facilities. It contains eleven civil general hospitals, and seven or eight special hospitals, exclusive of various charities for the relief of the aged, foundlings, orphans and the insane. All these institutions are under the direction of government; and clinical instruction is not only permitted, and, in every way, under proper regulations, facilitated, but it is made obligatory on the part of those of the hospital physicians and surgeons who are clinical professors in the university.

A very large proportion of the citizens of Paris, when attacked with disease, resort to the hospitals. During the year 1851 there were admitted into the general and special hospitals collectively, 85,760 patients, of which 61,005 were medical, and 24,755 surgical cases. Of this number 7,201 died. The proportion of fatal cases thus being not very far from one for every twelve persons admitted. The *hospices and maisons de retraite*, (sanitary retreats) in addition, received, during the same year, 9,290 cases. These facts certainly show an immense amount of clinical material. But the advantages to the medical student, and to the clinical observer, pertaining to so vast a field, are in many respects more apparent, than real. The visits at all the hospitals take place in the morning between the hours of seven and ten. The charge of the cases at each hospital is distributed among several medical and surgical officers, allotting to each, never more than a hundred beds. Of the diseases and operations, therefore, which are daily to be seen at the different hospitals, a student, each day, can only see, in connection with the medical or surgical officer in attendance, those which are presented in a collection of one hundred patients. He may gratify his curiosity by visiting, on different days, different hospitals, or different wards of the same hospital, but there is little advantage in this beyond the mere gratification of curiosity; and, in fact, they who wish to make the most profitable use of time usually select the service of some one surgeon, or physician, confining themselves, mainly, to his wards. In the range for selection, therefore; consists the chief advantage to be derived from the large number of hospitals which Paris contains. Moreover, the benefit derived from clinical studies depends not on a large number of cases, but on the use which is made of a few.

A hundred medical cases, in a general hospital, are more than a physician can properly examine, and divides too much the attention of the student. A distinguished teacher and author, who is, at this moment, working as effectively as any one in clinical studies, I refer to Dr. WALSHE, of London, has charge of but twenty beds. He declares that he cannot do justice to himself, his patients, and his classes, if this number be increased. And from this amount of material he has collected data for valuable statistical investigations, witness his reports on cancer, and pulmonary consumption, in addition to his excellent treatise on diseases of the lungs and heart. It is in the *special* hospitals that the advantages pertaining to the large clinical resources of Paris chiefly consist. Institutions restricted to cases of certain diseases, as for example, the St. Louis hospital devoted mainly to diseases of the skin, undoubtedly are of great advantages to persons desirous of making the study of these diseases a special pursuit. In this respect, probably no other city in the world affords equal opportunities. But so far as concerns the general objects of clinical researches and instruction, that is, for the study of miscellaneous diseases, an institution of the size of the Louisville Marine hospital is abundantly ample.*

The administration of public relief in Paris is admirably systematic and complete in all its details. Excepting accidents and urgent cases, patients apply for hospital aid at the "central bureau of public assistance." To this bureau are sent daily reports of the number of vacant beds in each hospital, and cases are examined and distributed among the different institutions by a medical officer in attendance for that purpose. In the intercourse of the medical and surgical officers with the patients I was struck with the manifestations of consideration and kindness, which were almost uniformly exhibited. In neatness and order few exceptions could be taken, although, in these, and all other particulars, well regulated American institutions are not inferior, and none of the haspitals which I visited in Europe, in elegance could be compared to the Massachusetts general hospital at Boston. The latter is probably the finest hospital in the world.

*I should remark that in making these statements I have in view more especially the study of diseases exclusive of surgical operations. The statements will not apply to the latter with the same degree of force.

It may be expected that I shall say something of French medical practice. The opinion generally obtains out of France, that therapeutics, in that country, receive very little attention. Some one has said that a fatal termination of disease is considered by the French practitioners to be an element of success in the management of cases, because the physician is then enabled, by an autopsy, to verify the correctness of his diagnosis! This, of course, is an humorous exaggeration; but it is unquestionably true that active remedial agents are employed less freely and uniformly than in American, and still more in English practice. The French physician is oftener satisfied to watch the progress of disease, and, relying on the recuperative power of nature, content himself with the regulation of diet and hygienic measures. If he err in carrying this system to an extreme, in certain cases, it must be admitted that the tendency, with English practitioners especially, and, to a greater or less extent, with many of those of our country, has been to an opposite extreme. Without discussing this point, which would be impossible on the present occasion, I will dismiss it with the remark that a *juste milieu*, a middle ground, is perhaps in general the most consistent with our present knowledge of the *ars medendi*. In not a few respects, I do not hesitate to express the belief, diseases are better managed by judicious American practitioners, than in Paris. We could certainly reciprocate the obligations we are under to the French by some valuable hints concerning the treatment of diseases; but such an interchance of commodities is not very easily effected. The French adopt practically the precept that it is better to give, than to receive. They are willing to supply oil to feed the lamps of others, but they do not conceive it possible for themselves to stand in any need of the reciprocity of such a favor. As respects the importation of scientific truth, our Gallic friends are nearly as isolated as if an embargo were placed on this kind of commerce. A small proportion only of their leading medical men are acquainted with the English language, and so imbued are they with the sentiment that scarcely anything of great value can emanate elsewhere than in *la belle France*, that they are slow to attach importance to aught that may be proffered to them from abroad. I should be sorry to do them injustice by these remarks, but I could not avoid such an impression as the result of my observations and inquiries.

An instance illustrative of the great simplicity of Frence therapeutics, with which both physician and patient is satisfied, was related to me by M. Bossange, a gentleman whose courtesy to Americans is well known to most of those who have visited Paris. He informed me that Dr. Capuron, a medical author of some merit, who died, at an advanced age, not long since, was for 20 years his family physician. During this period his children were born and reared, and numerous cases of illness occurred, but on no occasion could he recollect that he had procured aught from any apothecary save leeches. Aside from the latter, the remedies consisted wholly of domestic *tisanes*, conjoined with regulation of diet and regimen, and baths. In no instance had disease terminated fatally in his family, and he was very well contented with his choice of a physician.

M. Capuron had some excentricities which it may be interesting to mention, since his name has been introduced. He was what the French term a *vieux garcon*, in other words an old bachelor, and exceedingly economical. As an illustration of the latter trait, M. Bossange told me that once, or oftener, every year, he invited him to dine, and as he lived at a distance, and kept no riding establishment, he invariably came in a public carriage. As soon as he was admitted he requested the servant to pay the coachman for bringing him to the house. His frugality did not end here. After every such occasion he regularly entered a charge against his host for a professional visit; so that M. Bossange had not only to give his physician a dinner (which by the way in Paris is a dinner saved), but defray his travelling expenses, and pay the usual fee for a medical visit in the bargain! Nevertheless, with true French politeness, no exceptions were taken by the patient to the conditions upon which his hospitality was accepted, and the invitations were regularly made and duly honored for twenty years.

By his habits of accumulation, M. Capuron rolled up during a long life, three millions of francs. He was accustomed daily to visit some one of the hospitals, but finally settled on the service of Bouillaud, having arrived at the conclusion that he was the ablest of the faculty; and in testimony of his preference, he left at his death the bulk of his large property to the fortunate physician of *La Charité.*

The medical stranger in his prominades in Paris naturally looks around for the houses, or offices of his professional brethren. He looks in vain, howev.r, for any *insignia* by which he may distinguish them. With us, as is well known, a practitioner is sufficiently considerate of the public welfare to notify passers by, in letters so plain that he who runs may read, of the fact of their being in the immediate vicinity of places where medical and surgical aid may be obtained. No such philanthropic intimations are extended to the Parisians. They are obliged to find the residences of doctors as they best can, it being considered unprofessional, and disreputable, to put out signs, or even to display a modest door-plate. It is presumed that if a patient desires the services of a particular physician or surgeon, he will take pains to discover his place of abode by making inquiries, or resorting to the directory. It may be asked, "are the irregular practitioners guided by this rule of professional propriety?" A class of irregular practitioners, so far as I could learn, does not exist in France. The practice of medicine and surgery is confined to those legally authorized. The medical profession proper, consists alone of graduates of the University of France; but for those who are better satisfied with practitioners of an inferior education, a lower order is provided, called the officers of health, (*officiers de santé*). To obtain a license as an officer of health, certain attainments are required, tested by an examination, but much below those requisite for an university degree. The greater number of the health officers are distributed in the rural departments. The number registered in Paris in 1853 was only 179, the number throughout France for the same year being 7,126. The number of doctors in medicine in Paris, in the same year, was 1,337; the ratio of both orders of practitioners, collectively, to the population, being 1 for 763 inhabitants. This proportion is much less than in any of the cities of our country. In the departments the ratio is still smaller, being one practitioner for every 1,000 inhabitants. The explanation of the comparatively small number of medical practitioners in France, is to be found in the expense attending a medical education, taking into consideration the support for four years, in addition to the fees; and in the fact that the laws forbidding, under severe penalties, any but those legally authorized to practice, are likely to be enforced.

My discursive remarks, gentlemen, have already occupied so large a portion of the hour, that I must hasten to say a few words in conclusion, on a topic which I do not wish to pass by in silence. The question has been repeatedly put to me since my return, "well, what do you think of medical students or young physicians going to Paris for professional pursuits!" It was an object of interest with me to prepare myself to answer this question, and I avail myself of the present occasion to do so in a few words. In my opinion, the step is one of doubtful expediency for the following reasons:

The language is not a trifling obstacle in the great majority of cases. A knowledge of the French tongue sufficient for ordinary purposes may doubtless be attained by a moderate expenditure of time and labor; but a degree of proficiency by which it ceases to be not a small obstacle in the acquisition of accurate knowledge by means of oral instruction, demands more patience and application than most persons are willing to bestow.

Another source of difficulty, as well as danger, is the fact that a young man is placed in the midst of great advantages often without chart or compass to direct his steps. With no fixed direction of tastes or pursuits, not having before him any special objects, in many instances habits of application remaining to be formed, he is confused by the multiplicity of the opportunities by which he is surrounded, and in his perplexity perhaps selects no definite paths of study. Under these circumstances he does nothing beyond gratifying his curiosity, by seeing different men and institutions, which, if continued, tends to dissipation of the mind, instead of contributing to any solid improvement.

A greater objection still, relates to the temptations to pleasure and vice which meet him on every side. Removed so far from the influence of home and friends, and without the wholesome restraints of social associations or even public opinion, it demands greater stability of purpose, and moral strength than fall to the lot of many, to resist allurements which (aside from other pernicious effects) seduce the mind from its attachment to scientific pursuits.

These reasons for the opinion I have expressed are not based on theoretical ground. They are often enough illustrated, notwithstanding the numerous honorable instances in which they fail to be practically exemplified. The advantages, as a general remark, are not sufficient to render it good policy to incur the liabilities to disappointment. If time permitted, I should be glad to discuss, at some length, the several points which would be suggested by a comparison of the resources for medical pursuits which may be made available at home, with those which exist abroad, so far as my acquaintance with the latter extends. Such a discussion however, must be reserved for some other occasion, or postponed indefinitely. While I admit freely that in some important particulars we labor under present disadvantages in this country, they are not such as may not be overcome by individual energy and perseverance. It is a striking attribute of studies pertaining to the phenomena of life, in health and disease, that they have no necessary connection with time or place. External incentives, facilities and helps are useful and desirable, but a determined purpose, and persisting industry, may accomplish as much in the several departments of our science in America as in Europe. I should not have to go far to cite examples of this truth. A proper ambition and self-reliance, as individuals, together with concerted action, more mutual confidence and a spirit of independance in the American medical profession, as a body, are all that is necessary to effect objects which will make our country as distinguished for its contributions to the progress of medical science, as it is for what it has already done in behalf of civil and religious liberty.

*Gentlemen of the Medical Department of the University of Louisville:* As the organ of the Faculty on this occasion, I bid you welcome to this institution. I feel assured that I convey the sentiments of my colleagues when I say, that, in greeting you as pupils, we would receive you as friends, and, prospectively, brethren in the profession which we love and honor.

On our part we meet you with a determination to employ our best efforts to render your connection with the University of Louisville, in every respect, agreeable and profitable to yourselves. We look, on your part, for a disposition to avail yourselves, to the utmost, of the advantages which will be placed at your disposal.

Incited and guided by such sentiments, with the blessing of Divine Providence, we may engage in the labors upon which we are about to enter, with the confident assurance that our mutual hopes and efforts will be crowned with success.

---

NOTE.—After the foregoing address had been placed in the hands of the printer, I was favored with a copy of an introductory lecture delivered at the opening of the present session of the Jefferson Medical College, by Prof. ROBLEY DUNGLISON, in which are enunciated views concerning the propriety of going abroad for medical knowledge, somewhat similar, in their tone, to those which I have advanced. I cannot forbear expressing a sense of gratification that my conclusions on this subject are in accordance with those entertained by one so distinguished, and whose opinions on matters pertaining to medical education are entitled to the highest consideration. And I avail myself of this opportunity to acknowledge the pleasure and profit derived from the personal intercourse with Prof. DUNGLISON, which I was privileged to enjoy at Edinburgh, and during my return voyage across the Atlantic.

